# American *Aedes vexans* Mosquitoes are Competent Vectors of Zika Virus

**DOI:** 10.4269/ajtmh.16-0963

**Published:** 2017-06-07

**Authors:** Alex Gendernalik, James Weger-Lucarelli, Selene M. Garcia Luna, Joseph R. Fauver, Claudia Rückert, Reyes A. Murrieta, Nicholas Bergren, Demitrios Samaras, Chilinh Nguyen, Rebekah C. Kading, Gregory D. Ebel

**Affiliations:** 1 Arthropod-Borne and Infectious Diseases Laboratory, Department of Microbiology, Immunology and Pathology, Colorado State University, Fort Collins, Colorado

## Abstract

Starting in 2013–2014, the Americas have experienced a massive outbreak of Zika virus (ZIKV) which has now reached at least 49 countries. Although most cases have occurred in South America and the Caribbean, imported and autochthonous cases have occurred in the United States. *Aedes aegypti* and *Aedes albopictus* mosquitoes are known vectors of ZIKV. Little is known about the potential for temperate *Aedes* mosquitoes to transmit ZIKV. *Aedes vexans* has a worldwide distribution, is highly abundant in particular localities, aggressively bites humans, and is a competent vector of several arboviruses. However, it is not clear whether *Ae. vexans* mosquitoes are competent to transmit ZIKV. To determine the vector competence of *Ae. vexans* for ZIKV, wild-caught mosquitoes were exposed to an infectious bloodmeal containing a ZIKV strain isolated during the current outbreak. Approximately 80% of 148 mosquitoes tested became infected by ZIKV, and approximately 5% transmitted infectious virus after 14 days of extrinsic incubation. These results establish that *Ae. vexans* are competent ZIKV vectors. Their relative importance as vectors (i.e., their vectorial capacity) depends on feeding behavior, longevity, and other factors that are likely to vary in ecologically distinct environments.

## Introduction

Zika virus (ZIKV; *Flaviviridae*: *Flavivirus*) is a mosquito-borne virus currently spreading in the Western Hemisphere. It has now reached ≥ 49 countries and territories in the Americas including the United States and can cause febrile illness, severe neurological complications, and congenital malformations (e.g., microcephaly).^1^ *Aedes* (*Stegomyia*) *aegypti* Linnaeus and *Aedes* (*Stegomyia*) *albopictus* Skuse are the main mosquito vectors of ZIKV.^1^ Travel-associated cases have now been confirmed in all U.S. states and much of Europe (http://www.cdc.gov/zika/geo/active-countries.html). It is therefore important to define the competence of other potential ZIKV vectors that could feed on viremic individuals.

The inland floodwater mosquito, *Aedes* (*Aedimorphus*) *vexans* (Meigen), has a worldwide distribution and is known to aggressively bite humans.^2^,^3^ These mosquitoes are capable of transmitting West Nile virus, St. Louis encephalitis virus, Western and Eastern equine encephalitis viruses, Rift Valley fever virus (RVFV), and the dog heartworm parasite, *Dirofilaria immitis*.^4^,^5^ We therefore sought to determine the vector competence of field-caught *Ae. vexans* mosquitoes to transmit ZIKV after oral exposure.

## Materials and Methods

*Aedes vexans* mosquitoes were collected from August to early October 2016 using Centers for Disease Control and Prevention light traps baited with dry ice. Twenty traps were placed at 105°3 20.10 W, 40°34 44.27 N in Fort Collins, CO. Traps were placed on the nights of August 17, 23, and September 8, 2016. Traps were set at approximately 1,600 hours and were collected by 1,000 hours the following morning, at which point *Ae. vexans* females were identified morphologically.^3^ Mosquitoes were housed at 27°C, 80% relative humidity, and 16:8 (light/dark) photoperiod for no more than 7 days before further manipulation.

Vector competence experiments were performed as previously described.^6^ In brief, mosquitoes were given an infectious bloodmeal consisting of a 1:1 solution of cell culture supernatant containing freshly grown ZIKV and defibrinated calf blood. ZIKV strain PRVABC59 (accession no. KU501215) was freshly grown for each biological replicate by infecting semiconfluent monolayers of Vero cells at a multiplicity of infection (MOI) of 0.01 and harvesting virus 5 days postinfection.^7^ ZIKV titers in the resulting bloodmeals were determined by plaque assay (Table 1) according to standard methods.^8^ Three biological replicates were performed, hereafter referred to as BR1, BR2, and BR3 (Table 1). Fourteen days after feeding of the infectious bloodmeal, mosquitoes were cold-anesthetized, and legs, midguts, and salivary secretions were collected as described previously.^6^ Samples were preserved at −80°C until screening by plaque assay on Vero cells. Midguts shown to be virus positive indicated infection, virus-positive legs indicated dissemination, and saliva shown to be virus positive indicated transmission. Infection, dissemination, and transmission rates are defined as the proportion of mosquitoes with infectious virus in their midguts, legs, and saliva, respectively, out of the total number of blood-fed mosquitoes. All saliva samples shown positive for virus on plaque assays were confirmed to be ZIKV positive by quantitative reverse transcription polymerase chain reaction^9^ (data not shown).

## Results and Discussion

Infection rates ranged from 66% (38/58) in BR1 to 91% (53/58) in BR2, dissemination rates ranged from 3% (2/58) in BR1 to 25% in BR3 (8/32), and transmission rates ranged from 2% (1/58) in BR1 to 7% (4/58) in BR2 (Table 1). High variability between replicates is likely related to genetic variability between generations of wild *Ae. vexans* mosquitoes and/or environmental fluctuations (shortening of photoperiod, temperature changes, and precipitation) that were naturally occurring over the summer months.

To our knowledge, our study is the first to assess the vector competence of wild *Ae. vexans* mosquitoes for transmission of ZIKV. We demonstrated that wild-caught *Ae. vexans* from northern Colorado were highly susceptible to infection by ZIKV. However, dissemination and transmission rates were relatively low, indicating the existence of a moderately strong midgut escape and salivary gland barriers. More notably, however, was the low transmission rate compared with infection and dissemination rates. Since we did not collect salivary glands as part of this study, we cannot determine whether low transmission was due to infection of or egress from this tissue. Moreover, the data presented here indicate that *Ae. vexans* are susceptible to ZIKV infection, but virus transmission potential appears to be low. Additionally, it is important to note that mosquitoes were fed a relatively high bloodmeal concentration of ZIKV. Viremias greater than 7 log_10_ genomes/mL have been reported,^10^ which is considerably lower than what was used here. However, several previous reports have determined that mosquitoes are less susceptible to artificial-membrane infections as opposed to infection from viremic animals.^11^–^13^

Our observations, in addition to the wide distribution and aggressive biting nature of *Ae. vexans*, suggest this mosquito as an unlikely, but potential contributor to the spread of ZIKV in areas within and outside the geographical range of *Ae. aegypti* and *Ae. albopictus*. *Aedes vexans* was more competent at transmitting ZIKV than other North American species tested to date: *Culex pipiens quinquefasciatus* Say, *Culex pipiens pipiens* L., and *Culex tarsalis* Coquillett, and *Aedes* (*Protomacleaya*) *triseriatus* (Say) mosquitoes were shown to be highly refractory to infection by ZIKV.^6^,^14^,^15^ By comparison, infection, dissemination, and transmission rates in *Ae. aegypti* have been observed to be as high as 76.7% (23/30), 46.7% (14/30), and 10% (3/30) and 50% (15/30), 6.7% (2/30), and 3.3% (1/30) in *Ae. albopictus*^16^ (data modified from Chouin-Carneiro and others). Under certain situations, the moderate competence of *Ae. vexans* for transmission of ZIKV demonstrated in this study may be enough to contribute to local transmission. High population densities of poorly competent vectors have been observed to maintain virus transmission^17^ and such population densities have been shown to occur with *Ae. vexans*.^2^,^18^ Furthermore, the competence of *Ae. vexans* for transmission of RVFV was also demonstrated to vary substantially from incompetent (Colorado, California) to highly competent (Florida) areas, highlighting the importance of future studies to evaluate populations of *Ae. vexans* from multiple geographic areas.^19^,^20^ Finally, vector competence contributes relatively weakly to vectorial capacity, particularly when compared with the host biting rate and probability of daily survival.^21^ Moreover, the relative importance of *Ae. vexans* as vectors (i.e., their vectorial capacity) depends on feeding behavior, longevity, and other factors that are likely to vary locally and that powerfully impact the epidemiological significance of *Ae. vexans* mosquitoes as ZIKV vectors.

## Figures and Tables

**Table 1:** Competence of *Aedes vexans* mosquitoes as vectors of Zika virus, number of virus positive/number bloodfed (%; 95% CI)

Experiment	Infected	Disseminated	Transmitted	Bloodmeal titer	
				Genomes/mL	PFU/mL
BR1	38/58 (66)	2/58 (3)	1/58 (2)	4.15E + 10	7.00E + 06
BR2	53/58 (91)	14/58 (24)	4/58 (7)	3.05E + 10	1.30E + 07
BR3	27/32 (84)	8/32 (25)	2/32 (6)	2.76E + 10	1.70E + 07
Total	118/148 (80; 48.3 to 112.37)	24/148 (16; −13.53 to 48.19)	7/148 (5; −1.57 to 11.57)		

CI = confidence interval; PFU = plaque-forming units.
